# Seasonal development of a coastal microbial mat

**DOI:** 10.1038/s41598-019-45490-8

**Published:** 2019-06-21

**Authors:** Daniela Clara Cardoso, Mariana Silvia Cretoiu, Lucas J. Stal, Henk Bolhuis

**Affiliations:** 1 Department of Marine Microbiology and Biogeochemistry, Royal Netherlands Institute for Sea Research, and Utrecht University, Den Hoorn, The Netherlands; 2 Freshwater and Marine Ecology (IBED-FAME), University of Amsterdam, Amsterdam, The Netherlands; 3 Present Address: Bigelow Laboratory for Ocean Sciences, East Boothbay, ME 04544 USA

**Keywords:** Ecosystem ecology, Microbial ecology

## Abstract

Growth and activity of coastal microbial mats is strongly seasonal. The development of these mats starts in early spring and fully maturate during late summer, where after growth ceases and subsequently the mat deteriorates by erosion and decomposition in winter. Here, the composition of the microbial community of three different mats developing along the tidal gradient of the North Sea beach of the Dutch barrier island Schiermonnikoog was analysed. The 16S ribosomal RNA molecules and the associated gene were sequenced in order to obtain the active (RNA) and resident (DNA) community members, respectively. Proteobacteria, Cyanobacteria, and Bacteroidetes dominated the mats during the whole year but considerable differences among these groups were found along the tidal gradient and seasonally when observed at a finer taxonomic resolution. Richness and diversity increased during the year starting from a pioneering community that is gradually succeeded by a more diverse climax community. The initial pioneers consisted of the cold-adapted photoautotrophic cyanobacterium *Nodularia* sp. and potential cold adapted members of the alphaproteobacterial *Loktanella* genus. These pioneers were succeeded by, amongst others, cyanobacteria belonging to the genera *Leptolyngbya*, *Lyngbya*, and *Phormidium*. At the upper littoral (Dune site), which was characterized by an extensive salt marsh vegetation, the mats contained a distinct bacterial community that potentially contribute to or benefit from plant decay. This study reports in detail on the seasonal changes and succession of these coastal microbial mat communities and discusses the potential forces that drive these changes.

## Introduction

A fundamental question in microbial ecology is how community composition changes in response to temporal and spatial variations^1^. Sampling a microbial community at one time point and at one location gives only a snapshot and may underestimate the actual diversity and composition and obviously misses out changes therein. It is well-known that benthic microbial ecosystems are characterized by spatial heterogeneity that is generated by small-scale variations of physicochemical conditions or just by chance^2^. Different physicochemical parameters may also determine the differences in community composition at a larger spatial scale. During a year, succession may change the composition of the microbial community^3^. In phototrophic microbial mats, seasonal variations in temperature, day-length, incident sunlight angle, and daily photon irradiation determine the success of individual members of that community. Daily variations such as the day-night cycle and the tides in coastal microbial mats are unlikely to influence their community composition but they may determine whether an organism becomes active. The same holds for physicochemical parameters^4^. It is therefore more informative to analyze both the active and resident members of a microbial community^5^.

Microbial mats are considered to be modern equivalents of Precambrian stromatolites, the oldest ecosystems known from the fossil record^6^. Microbial mats are globally widely distributed and are well-documented from coastal areas and extreme environments such as intertidal sediments^7^, hot springs^8^, Antarctic ponds^9^ and hypersaline lakes and ponds^10^. Several studies have shown that microbial mats and especially those developing on coastal intertidal sediments are amongst the most diverse ecosystems known, composed of hundreds of different species of bacteria that reside in the top 5 mm of the sediment^11^. Microbial mats appear to be stable ecosystems that are resilient to the large physicochemical fluctuations to which they are exposed, while maintaining the characteristic structure of vertically stratified functional groups of microorganisms^12^. Coastal microbial mats are exposed to highly dynamic conditions that may change on timescales of minutes causing steep and fluctuating gradients within the sediment. Changes in light intensity, temperature, salinity, pH, inorganic carbon, oxygen, sulfide, and other physicochemical parameters lead to the development of a highly diverse microbial community that allows it to respond in an adequate way^13^. However, little is known about spatio-temporal changes of the microbial mat community composition and their active components.

In a previous study an attempt was made to analyze the community composition in the same microbial mat system^11^. That study was limited in sampling size and considered only the ~60 nt V9 region of the 16S rRNA gene using the Life Science 454 pyrosequencing technique. Moreover, only the DNA derived resident community was analyzed in that study.

The present study aimed to unravelling the community composition and their active components of three different microbial mats along the tidal gradient as well as the succession from spring to autumn, taking multiple samples per site, using Illumina based high throughput amplicon sequencing of the V3-V4 16S rRNA and its corresponding gene fragment. In addition, we determined the occurrence of specific cyanobacterial ecotypes and their spatio-temporal distribution. A description of the succession in the community is given and interpreted in terms of the annual cycle of microbial mat formation and destruction^14^.

## Material and Methods

### Sampling procedure

**Figure 1:**
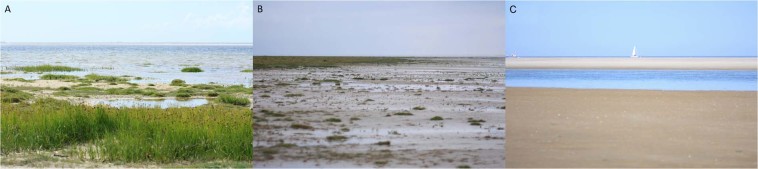
Site images of the microbial mats from the Dutch barrier island, Schiermonnikoog. (**A**) Dune station, (**B**) Intermediate station, (**C**) Tidal station.

Samples were taken from microbial mats developing on the North Sea beach of the Dutch barrier island Schiermonnikoog in November 2013 (autumn), May 2014 (spring), and August 2014 (summer). Sampling was performed at roughly 4-h intervals during a 24-h period. Different mat types developed on the beach perpendicular to the dunes along the tidal gradient that represented a natural salinity gradient. The three stations (here indicated as Tidal, Intermediate, and Dune) were established and sampled for previous studies^15,16^. The Dune station (53°29′25.0″N 6°08′40.0″E) is located close to the Dunes and was characterized by an abundant salt marsh vegetation (Fig. 1A). These mats are rarely inundated by seawater and receive mainly freshwater from rain and upwelling groundwater. The Intermediate station (53°29′27.0″N 6°08′32.9.0″E) is regularly inundated but is most of the time exposed and therefore influenced by both seawater and rain (Fig. 1B). The Tidal station (53°29′31.9″N 6°08′32.23.9″E) is situated near the low water mark and is frequently inundated with seawater. Vegetation was absent from this station (Fig. 1C). The tidal mats are diatom dominated communities that are embedded in copious amounts of extracellular polymeric substances (EPS). The tidal mats are exposed to physical forces produced by the tidal currents and are devoid of the macroscopically visible (multi-coloured) layers of functional groups of microorganisms typical of the other mats on the beach. The intermediate mats are classical multi-coloured, layered mats dominated by cyanobacteria. These mats develop early spring and eventually produce rigid structures that are often destructed due to erosional events in the winter. The Dune station is the vegetated zone (salt marsh) of the beach that has extended its area towards the sea over the past 30 years^17,18^.

From each of the three mat types, 5 samples were taken within an approximately 15 × 15 m area and this was repeated for the three seasons of interest resulting in a total of 45 samples. The top 5 mm of the intermediate and dune mats containing the actual cyanobacterial mat and the upper part of the permanent anoxic black layer was sampled using sterile 10-ml syringes from which the top was removed. The same depth was sampled from the tidal station. Samples were put in sterile 15-ml Falcon tubes containing 2.5 ml of LifeGuard^®^ Soil Preservation Solution (MOBIO, Carlsbad, CA, USA), mixed and immediately flash frozen in liquid nitrogen. For transfer to the laboratory the samples were placed on dry ice and upon arrival stored at −80 °C until nucleic acids were extracted.

### Nucleic acids extraction and cDNA synthesis

Before nucleic acid extraction, the LifeGuard® solution was removed by centrifugation (5 min at 10,000 rcf) and pipetting off the liquid phase. Nucleotides were extracted from approximately 1 g of mat material from each sample. DNA and RNA were co-extracted from the same sample using RNA PowerSoil® Total Isolation Kit in combination with the DNA elution accessory kit (MoBio Laboratories, Carlsbad, CA) according to the manufacturer’s instructions. The DNA yield and concentration were checked by using a NanoDrop 1000 photometer (NanoDrop, Wilmington, DE, USA) and molecular weight estimated by agarose gel electrophoresis. The 45 DNA extracts were normalized to 20 ng DNA/µl, stored at −80 °C until sent for amplicon sequencing. The quality and quantity of the RNA was checked on an Agilent 2100 Bioanalyzer (Agilent Technologies, Santa Clara, CA, USA) with the Agilent RNA 6000 Nano Kit. Extracts with an RNA Integrity Number (RIN) lower than 6 were discarded resulting in the loss of one sample for DNA analysis and two samples for RNA analysis. Possible DNA contamination was removed by treating the RNA extract with DNase using TURBO DNA-free™ DNAse kit (Ambion/Life Technologies - Thermo Fisher Scientific, USA) according to the manufacturer’s instructions. Failure to PCR amplify a 16S rRNA gene product using universal bacterial primers B8F (forward)^19^ and U1492R (reverse)^20^ and agarose gel electrophoresis (1.5% agarose gel) was considered as sufficient indicative for the complete removal of residual DNA from the RNA fraction. Subsequently, complementary DNA (cDNA) was synthetized using SuperScript® III Reverse Transcriptase (ThermoFisher Scientific, Pittsburgh, PA, USA) according to the manufacturer’s instructions. The successful reverse transcription of the RNA into cDNA was confirmed by PCR as described above and was shown by the presence of a band after agarose gel electrophoresis. The 45 cDNA samples were stored at −80 °C until sent for sequencing. 16S rRNA gene amplicon sequencing was carried out by BGI (http://www.genomics.cn/en/index, China) on the Illumina MiSeq platform (PE300) providing an approximately 465 nt long fragment after pairing with a 135 nt overlap. Based on previous analysis, the V3-V4 region of the 16S rRNA was chosen for amplification in order to provide good coverage^14^. The primers used were: V3 primer – 338F 5′-ACTCCTACGGGAGGCAGCAG-3′ ^21^ and V4 primer – 806R 5′-GGACTACHVGGGTWTCTAAT-3′ ^22^. Library construction, barcoding and amplicon sequencing were all performed by BGI (https://www.bgi.com/). Sequences have been deposited at the NCBI small read archive under accession number PRJNA503134.

### Bioinformatics and statistical analysis

Sequence analysis was done using the QIIME bioinformatics pipeline^23^. High quality reads were merged with their pairs using PEAR^24^, while reads with multiple ambiguous bases, composed by less than 250 nucleotides or when the average quality scores using a sliding window of 40 bp dropped below a satisfactory threshold of 25 Phred were excluded. Suspected chimeric sequences were removed using Vsearch version 1.1.3 (https://github.com/torognes/vsearch). Subsequently, OTUs were clustered at 95% sequence identity. The cluster centroid for each OTU was chosen as the representative sequence for taxonomic assignment using the RDP Bayesian Classifier^25^ against the SILVA SSU non-redundant database (release 132) with a consensus confidence threshold of 0.8. OTU abundances are expressed as percentage of total reads per sample. To determine the sequencing depth for each sample, a rarefaction table was calculated. Microbial diversity between samples (beta diversity) and significance thereof was evaluated using non-metric multidimensional scaling (NMDS) on the Bray-Curtis dissimilarity matrix of the OTU table and permutation based-ANOVA (PERMANOVA and R vegan function adonis) using the vegan R Package^26^.

### Cyanobacterial ecotype analysis

Cyanobacterial ecotypes were defined by applying the same pipeline described above but clustering cyanobacterial OTUs at 100% identity. This was done using only the forward reads because they had less low-quality base calls compared to the reverse reads resulting in more statistically relevant numbers of reads per OTU. OTUs were aligned using Clustal Omega^27^ that also created a percent identity matrix between each sequence. A heat map of the identity matrix assisted the selection of OTU clusters with at least 95% sequence identity of the 275-nucleotide long region and these were considered ecotypes. A maximum likelihood tree of the alignment was generated with the software package RAxML^28^ using 500 bootstrap iterations. The phylogenetic tree was visualized using the online tool “ITOL” (Interactive tree of live; https://itol.embl.de/)^29^.

## Results

### Diversity and seasonal development of the microbial mat community

Sequence analysis of the V3-V4 region of bacterial 16S rRNA genes from the three mat types that were sampled at three different seasons revealed 977 unique OTUs with at least 95% sequence identity. In order to describe the richness and diversity within one mat type per season the relative proportion of OTU from each sample per station were averaged and the observed (Sobs) and estimated richness (Chao1) were calculated (Table 1). The observed richness covered 85–96% of the estimated Chao1 richness. The lowest values agreed with rarefaction curves not yet reaching an asymptote (data not shown). Among the DNA samples, the highest estimated richness was found in the Tidal station in autumn (910 OTUs) while the lowest number of OTUs was observed in the Dune station in spring (638 OTUs). In the RNA-derived dataset, in total 963 unique OTUs were identified, 14 less than in the DNA fraction. Lowest and highest estimated richness in the RNA samples were observed in the Dune station in spring (619 OTUs) and in autumn (884 OTUs), respectively (Table 1). For both DNA and RNA, the average estimated richness was higher in autumn when compared to spring. A less clear pattern emerged when the three mats were compared. Only the RNA derived dataset showed a slight increasing estimated richness along the tidal gradient from the Dune to the Tidal station, except in autumn when richness was highest in the Dune station and lowest in the Tidal station.

**Table 1:** General statistics based on 95% OTUs and Silva taxonomic assignments at the genus level.

Origin	Season	Type	#Reads after QC	Chao1	Shannon
Dune	Spring	DNA	11742	638	3,73
Inter			24749	799	4,17
Tidal			17427	727	4,55
Dune	Summer		19762	828	4,23
Inter			25342	805	4,62
Tidal			27279	824	4,84
Dune	Autumn		19984	888	5,03
Inter			30799	902	4,55
Tidal			22793	910	3,71
Dune	Spring	RNA	11742	619	3,12
Inter			75583	715	3,57
Tidal			52962	730	2,93
Dune	Summer		41301	630	3,53
Inter			115807	719	3,50
Tidal			27279	810	4,46
Dune	Autumn		38404	884	4,51
Inter			11235	757	4,19
Tidal			7406	654	2,51

The Shannon diversity estimator varied from 5.03 (Dune autumn) to 3.71 (Tidal autumn) in the DNA fraction and from 4.51 (Dune autumn) to 2.51 (Tidal autumn) in the RNA fraction (Table 1). An overall lower diversity was found in the RNA fraction (on average 3.59 compared to 4.38 in the DNA fraction). The diversity in both the DNA and RNA samples was on average highest in summer and lowest in spring. Spatial trends among the DNA samples revealed the lowest diversity in the Dune station and the highest diversity in the Tidal station, except in autumn when the trend was opposite. For the RNA fraction, a similar trend was only observed in autumn where diversity decreased from the Dune to the Tidal station.

### Non-linear multidimensional scaling analysis of spatial and seasonal variations of communities

**Figure 2:**
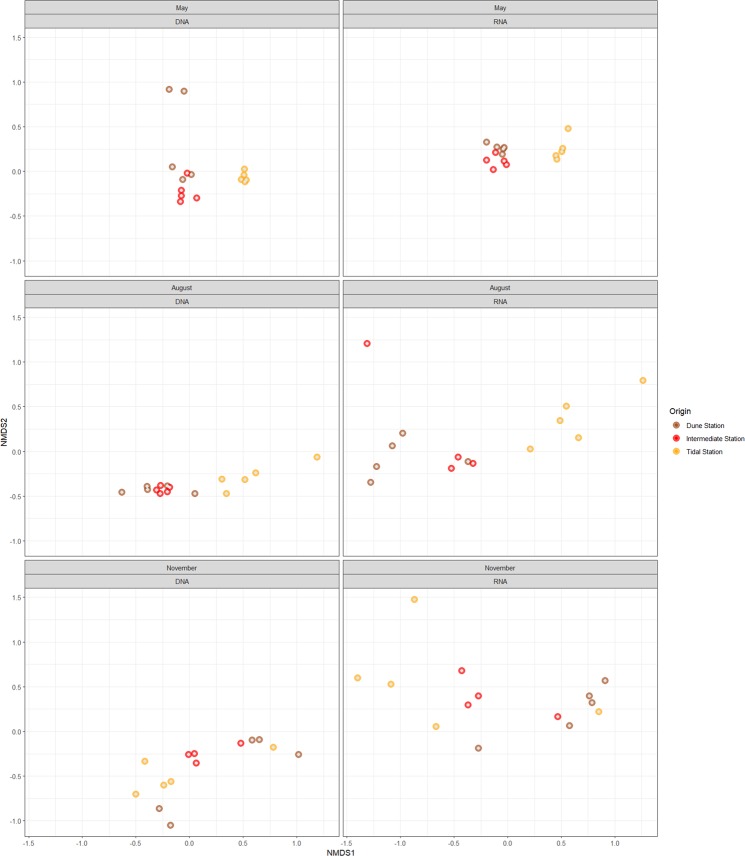
NMDS analysis of community dissimilarities (Bray-Curtis) between samples from the DNA fraction (left column) and RNA fraction (right column) divided over the three sampling seasons. Samples taken at the same location at the same time are indicated by the same colour.

In order to examine OTU-environment relationships, non-linear multidimensional scaling (NMDS) was performed using a Bray-Curtis dissimilarity matrix generated from the OTU relative proportion table that included all samples. For clarity, the resulting clustering was divided per nucleic acid source and per season (Fig. 2). This analysis shows that in spring and summer the differences in DNA derived community composition between samples from the Dune and Intermediate station was small and formed a distinct cluster relative to the Tidal station samples. This distinction between the Tidal station with the other two stations was also observed for RNA derived fraction in spring. In summer and autumn, the spread amongst the five samples per station of the DNA fraction was smaller compared to that of the RNA fraction. The differences in OTU composition as deduced from the spread within the station samples was lowest in spring, especially in the RNA fraction (Fig. 2). Notable, samples from the Intermediate mats were less divergent than those of the other two stations (Fig. 2). PERMANOVA analysis of the observed variance was performed on the complete dataset to uncover potential drivers of the observed diversity. The community composition of the three stations during the three seasons differed significantly from each other (*p* = 0.001) showing that both space and time affect the microbial community composition. Community composition derived from the DNA and RNA fraction also differed significantly (*p* = 0.001), showing a clear distinction between the resident and active fraction.

### Taxonomic identification of the community members

**Figure 3:**
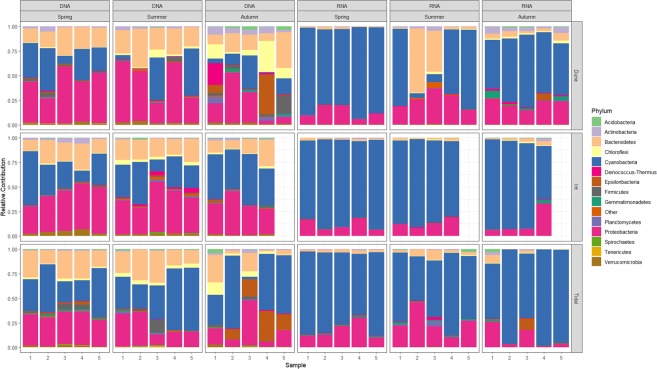
Relative contribution of the bacterial phyla. Sampling type (RNA, DNA) and sampling season are indicated on top. The sampling station is indicated in the right-side bar. Samples taken from the same station at the same time are indicated in the horizontal axis.

Taxonomic analysis assigned the 977 OTUs to 29 phyla and 501 genera. Proteobacteria (32%) were slightly more abundant than Cyanobacteria (31%) in the overall DNA derived dataset and were followed by Bacteroidetes at 19%. Together these three phyla represent more than 82% of the total community (Fig. 3). Throughout the year, Cyanobacteria were relatively more abundant in the Tidal mat (41%) and less abundant in the Dune mat (20%). In contrast, Proteobacteria were more abundant in the Dune station (36%) and less in the Tidal station (23%). Bacteroidetes ranged from 18% in the Intermediate station to 21% in the Dune station. Measured over the seasons, Cyanobacteria were distributed ranging from 30% in spring to 33% in autumn. In the Dune station, the relative contribution of the Cyanobacteria decreased from 28% to 13% from spring to autumn, while in the Tidal station the relative contribution increased with the season from 34% to 46%. Proteobacteria were slightly more abundant in spring (38%) and lower in autumn (24%), irrespective of the station. Bacteroidetes were also lowest in autumn (15%). Noteworthy was the large contribution of Chloroflexi in the Dune station (5%) relative to the Intermediate (3%) and Tidal stations (1%). Actinobacteria, Deinococcus-Thermus and Firmicutes were also most abundant in the Dune station relative to the other stations, while Verrucomicrobia were most abundant in the Intermediate station and lowest in the Tidal station. Epsilonproteobacteria (now proposed to be removed from the Proteobacteria and to form an independent phylum ‘Epsilonbacteraeota’^30^) were most abundant in autumn (8.8% of the total community) and found in low relative proportion in spring and summer (0.6 and 0.3%, respectively). This group comprised 5%, 4%, and 0.3% of the phyla in the Tidal, Dune, and Intermediate stations, respectively (Fig. 3). For the all-stations-combined abundant phyla, an increase was observed for Chloroflexi, Deinococcus-Thermus, Acidobacteria, Firmicutes, Fusobacteria, and Nitrospirae during the year. Verrucomicrobia decreased during the year. The autumn samples from the Dune station differed from the samples from all other stations and seasons by the higher contribution (20%) of the phyla Chloroflexi, Actinobacteria, Firmicutes, Deinococcus-Thermus, Planctomycetes, Acidobacteria, Verrucomicrobia, and Epsilonproteobacteria, which otherwise occur in low relative proportion (Fig. 3).

The RNA-derived dataset of inferred active bacteria was largely dominated by Cyanobacteria (73%). Proteobacteria and Bacteroidetes constituted 17% and 5%, respectively. Gemmatimonadetes, ranking at the 14^th^ position in relative proportion in the DNA fraction (0.4%), ranked at the 5^th^ position in the RNA fraction (0.7%). Cyanobacterial RNA reads were most abundant in the Intermediate station (84%) and in spring (82%), while the relative contribution of active Proteobacteria was highest in the Dune and Tidal stations (17.1% and 17.4%, respectively) and in summer (21%). Active Actinobacteria, Gemmatimonadetes, Epsilonproteobacteria, and Deinococcus-Thermus were lowest in the Intermediate station. Relative contributions of active Planctomycetes, Acidobacteria, and Firmicutes were largest in the Tidal station. Seasonal succession of the active populations was observed for Actinobacteria, Gemmatimonadetes, Chloroflexi, Deinococcus-Thermus, and Acidobacteria that increased in relative proportion from spring to autumn (Fig. 3).

Alphaproteobacteria were the most dominant proteobacterial class in the DNA fraction followed by the Gammaproteobacteria, which, according to the Silva database, is now proposed to include the former class of Betaproteobacteria as a novel order termed Betaproteobacteriales, and the Deltaproteobacteria (Supplementary Fig. 1). Alphaproteobacteria was the most abundant class in the Intermediate station and lowest in the Tidal station. Also, Alphaproteobacteria showed a decrease in relative contribution from spring to autumn while Gamma- and Deltaproteobacteria peaked in autumn. In the RNA fraction, all Proteobacterial classes were relatively less represented compared to the DNA fraction but ranked in the same order of relative proportion. Alphaproteobacteria were generally more abundant than other Proteobacteria classes. Active Gamma- and Deltaproteobacteria were lowest in relative proportion in the Intermediate station and highest in autumn.

### Genus level diversity

In total, 501 different genera were detected in the coastal microbial mats. The mats were dominated by the alphaproteobacterium *Loktanella*, which accounted on average for nearly 8% of the DNA derived sequence reads (Table 2). This genus was followed in relative proportion by the Cyanobacteria *Nodularia* (6.1%), by *Coleofasciculus PCC-7420* (5.2%), by cyanobacterial genus *Nodosilinea PCC-7*10*4* and by a non-assigned representative of the alphaproteobacterial family Rhodobacteraceae. Dominant genera from the resident fraction ordered per station and season are given in Tables 2 and 3. *Loktanella* was the dominant genus in the Dune station and its relative contribution decreased from 10.6% to 4.2% towards the Tidal station (averaged per season). Other genera, besides Cyanobacteria, that were more frequently found in the freshwater Dune to brackish Intermediate stations consisted of a novel genus belonging to the family of Rhodobacteraceae. Genera with a preference for the more marine part of the beach were *Coleofasciculus Leptolyngbya*, and *Sulfurimonas*, while *Roseovarius*, *Phormidium*, *Trichodesmium*, and *Rivularia* preferred the brackish Intermediate station (Table 3). In total, 189 genera preferred a marine environment (increasingly higher relative proportion from the Dune to Tidal station), while 140 genera were more abundant in the freshwater dominated Dunes station (data not shown). Out of 501 genera, 172 had the highest relative proportion in the Intermediate station.

**Table 2:** Abundant DNA derived (resident) genera grouped per season.

Spring	%	SD	Summer	%	SD	Autumn	%	SD
*Nodularia*	16.36	8.39	*Loktanella*	7.75	6.50	*Coleofasciculus*	14.03	25.08
*Loktanella*	12.00	6.19	*Leptolyngbya*	6.13	9.47	*Sulfurimonas*	8.06	12.05
Rhodobacteraceae_*g*	3.30	1.47	*Nodosilineaceae_g*	4.29	5.82	*Nodosilinea*	4.84	5.73
*Lyngbya*	2.89	3.85	*Rhodobacteracea_g*	4.20	2.47	*Loktanella*	3.69	3.27
*Nodosilinea*	2.75	2.46	*Nodosilinea*	3.31	3.21	Nodosilineaceae*_g*	2.48	4.71
*Roseovarius*	2.59	1.92	*Roseovarius*	3.18	3.42	Rhodobacteraceae*_g*	2.05	1.97
*Algoriphagus*	2.39	1.11	*Psychroflexus*	2.90	2.50	*Truepera*	2.04	5.57
*Bizionia*	2.30	1.67	*Trichodesmium IMS101*	2.64	3.32	*Rivularia PCC-7116*	1.84	2.78
*Congregibacter*	2.14	1.45	*Coleofasciculus*	2.14	2.57	*SBR1031_f_g*	1.65	4.60
Flavobacteriaceae_*g*	1.81	1.28	*Halomicronema*	1.93	2.01	Prolixibacteraceae*_g*	1.60	2.55
Cyclobacteriaceae_*g*	1.64	1.74	Desulfobulbaceae*_g*	1.63	2.16	*Phormidesmis*	1.59	1.82
*Roseicyclus*	1.55	0.80	*Halochromatium*	1.50	1.32	*Roseicyclus*	1.35	1.80
*Maribacter*	1.41	0.78	*Phormidium*	1.35	1.32	*Ilumatobacter*	1.28	1.21
*Lewinella*	1.39	0.64	*Roseicyclus*	1.21	0.85	A4b*_g*	1.16	1.99
Desulfobulbaceae*_g*	1.28	0.69	Flavobacteriaceae*_g*	1.20	1.47	*Roseovarius*	1.12	1.87

The replicates were averaged and are followed by the standard deviation (SD). f and g means non assigned family and genus.

**Table 3:** Abundant DNA derived (resident) genera grouped per station.

Dune	%	SD	Intermediate	%	SD	Tidal	%	SD
*Loktanella*	*10*.60	8.50	*Loktanella*	9.01	4.72	*Coleofasciculus*	12.54	24.74
*Nodularia*	5.43	8.20	*Nodularia*	6.64	9.82	*Nodularia*	6.33	8.67
*Sulfurimonas*	3.22	9.84	Rhodobacteraceae_*g*	5.11	1.87	*Leptolyngbya*	5.43	9.74
Rhodobacteraceae_*g*	3.13	1.97	Nodosilineaceae_*g*	4.75	5.14	*Nodosilinea*	4.91	3.29
*Roseovarius*	2.37	2.23	*Nodosilinea*	4.11	5.08	*Sulfurimonas*	4.62	8.23
*Lyngbya PCC-7419*	2.23	3.90	*Roseovarius*	3.96	3.35	*Loktanella*	4.19	2.93
*Congregibacter*	2.14	1.65	*Trichodesmium*	2.75	3.35	Cyclobacteriaceae_*g*	2.03	1.75
Nodosilineaceae_*g*	2.05	5.34	*Lyngbya*	2.40	1.39	Rhodobacteraceae_*g*	1.52	0.90
*Truepera*	1.89	5.41	*Rivularia*	2.26	2.46	Roseicyclus	1.42	1.18
*Nodosilinea PCC-7*1*04*	*1*.83	3.02	*Coleofasciculus*	1.73	1.80	Desulfobulbaceae_*g*	1.34	1.30
*Bizionia*	1.79	1.91	Flavobacteriaceae_*g*	1.67	1.49	Anaerolineaceae_*g*	1.33	2.05
Desulfobulbaceae_*g*	1.76	2.07	*Phormidium*	1.66	1.55	*Psychroflexus*	1.16	2.21
Flavobacteriaceae_*g*	1.62	1.73	Nostocaceae_g	1.39	1.08	*Phormidesmis*	1.14	1.18
Prolixibacteraceae_*g*	1.60	2.53	*Roseicyclus*	1.34	0.70	*Halomicronema*	1.12	1.97
SBR1031_f_*g*	1.54	4.46	*Phormidesmis*	1.31	1.72	Burkholderiaceae_*g*	1.04	1.10

The replicates were averaged and are followed by the standard deviation (SD). f and g means non assigned family and genus.

Seasonal succession was observed in 165 genera that increased in relative contribution from spring until autumn while 129 genera decreased during the course of the year. Of the dominant fractions (genera occurring at more than 1%), *Coleofasciculus* increased from 0.1% in spring to 14% relative contribution in autumn (Supplementary Table S1). Genera that increased in relative proportion with the seasons included two uncultured genera belonging to Cloroflexi (SBR1031), as well as *Sulfurimonas*, *Nodosilinea*, *Truepera*, *Phormidesmis*, and *Rivularia*. The opposite trend was also observed. Notably the most abundant genus in all three stations in spring, *Nodularia* (16.4%), was in low relative proportion in summer (0.7%) and autumn (0.9%). The abundant genera *Nodularia*, *Loktanella*, *Lyngbya*, and *Algoriphagus* were also highest in spring and lowest in autumn. Some genera were especially high in summer, among which were *Nodosilineaceae*, *Rhodobacteraceae*, *Leptolyngbya*, *Trichodesmium*, and *Roseovarius* (Supplementary Table S1).

Throughout the year and stations, the RNA fraction of the samples was dominated by *Coleofasciculus* followed by *Nodularia* and *Lyngbya* (Tables 4 and 5). Other abundant cyanobacteria included *Trichodesmium* and a representative of the Nodosilineaceae. Apart from the Cyanobacteria, reads belonging to *Aureispira* (Bacteroidetes), *Sulfiromonas* (Epsilonproteobacteria), *Halochromatium*, and *Roseovarius* were abundantly present in the RNA fraction. Particularly *Algoriphagus* reads were highly abundant in the summer-Dune sample and represented 19.3% of the total. Other genera were more evenly distributed over the stations and seasons (Table 5). Seasonal increase within the active population with a maximum relative contribution in autumn was found for *Sulfurimonas*, *Phormidesmis* and *Coleofasciculus* (Supplementary Table S2). Seasonal decrease was most prominent for *Nodularia*, a genus of the family Nostocaceae and *Loktanella*.

**Table 4:** Abundant RNA derived (resident) genera grouped per season.

Spring	%	SD	Summer	%	SD	Autumn	%	SD
*Nodularia*	36.70	19.88	*Coleofasciculus*	15.95	16.61	*Coleofasciculus*	32.80	34.09
*Lyngbya*	16.62	16.53	*Trichodesmium*	13.64	17.52	*Nodosilinea*	7.52	8.90
*Trichodesmium*	6.26	8.15	*Aureispira*	6.88	17.59	*Lyngbya*	7.10	7.30
Nostocaceae_*g*	4.01	2.89	*Nodularia*	6.64	15.06	*Rivularia*	3.26	5.39
Nostocales_f_*g*	3.09	3.26	*Lyngbya*	3.84	3.92	Nodosilineaceae_*g*	3.09	6.39
*Nodosilinea*	2.65	2.79	Oscillatoriaceae_*g*	3.68	10.42	*Nodularia*	2.77	3.51
*Coleofasciculus*	2.33	3.85	*Leptolyngbya*	3.61	6.02	*Phormidesmis*	1.81	1.77
Oscillatoriaceae_*g*	2.28	2.51	Nodosilineaceae_*g*	3.07	4.10	Nostocales_f_*g*	1.60	2.15
*Loktanella*	1.63	0.82	*Nodosilinea PCC-71*0*4*	2.84	4.09	*Ilumatobacter*	1.56	1.77
*Rivularia*	1.62	1.82	*Roseovarius*	2.51	2.77	Oscillatoriaceae_*g*	1.41	1.97
*Halochromatium*	1.55	1.87	*Halochromatium*	1.98	2.82	*Leptolyngbya*	1.37	1.43
*Roseovarius*	1.15	0.81	Rhodobacteraceae_*g*	1.75	0.95	*Gemmatimonas*	1.24	1.90
Rhodobacteraceae_f_*g*	1.11	0.41	*Loktanella*	1.52	1.41	*Sulfurimonas*	1.23	2.97
*Oscillatoria*	0.74	0.58	Oxyphotobacteria Incertae Sedis_f_*g*	1.01	1.80	*Cyanobium*	1.13	1.51
*Roseicyclus*	*0*.72	0.59	*Roseicyclus*	1.01	0.83	Burkholderiaceae*_g*	1.04	1.70

The replicates were averaged and are followed by the standard deviation (SD). f and g means non assigned family and genus.

**Table 5:** Abundant RNA derived (active) genera grouped per station.

Dune	%	SD	Intermediate	%	SD	Tidal	%	SD
*Nodularia*	16.12	17.22	*Coleofasciculus*	19.15	15.19	*Nodularia*	25.06	26.65
*Lyngbya*	14.15	15.05	*Lyngbya*	16.22	12.57	*Coleofasciculus*	24.08	35.86
*Nodosilinea*	8.30	10.16	*Trichodesmium*	11.30	11.92	*Nodosilinea*	4.89	4.04
*Coleofasciculus*	6.09	11.12	*Nodularia*	11.14	15.06	*Leptolyngbya*	3.42	5.92
*Trichodesmium*	5.89	9.46	Nodosilineaceae_*g*	5.13	6.39	*Lyngbya*	2.30	3.45
Nostocales_f_*g*	3.94	3.41	*Rivularia*	4.81	5.13	Nostocaceae_*g*	1.59	1.21
Oscillatoriaceae*_g*	3.13	2.52	Nostocaceae_*g*	2.79	3.76	*Halochromatium*	1.39	2.40
Nostocaceae_*g*	2.20	1.47	*Nodosilinea*	2.11	2.55	Rhodobacteraceae*_g*	1.10	0.76
Ilumatobacter	1.78	1.96	*Roseovarius*	2.08	2.29	Oxyphotobacteria Incertae Sedis_f_*g*	1.08	1.10
*Halochromatium*	1.67	2.05	Nostocales_f_*g*	1.71	2.28	*Roseicyclus*	1.06	0.83
*Loktanella*	1.55	1.11	Oscillatoriaceae_*g*	1.34	1.93	*Rubribacterium*	0.98	0.88
*Phormidesmis*	1.45	1.76	Rhodobacteraceae_g	1.04	0.48	*Synechocystis*	0.94	1.09
*Cyanobium*	1.34	1.69	*Halochromatium*	1.02	1.26	Burkholderiaceae_*g*	0.89	1.03
*Gemmatimonas*	1.30	2.11	*Leptolyngbya*	1.00	1.33	RD017_f_*g*	0.89	1.81
Rhodobacteraceae_*g*	1.13	0.39	*Loktanella*	0.90	0.62	Nostocales_f_*g*	0.80	1.22

The replicates were averaged and are followed by the standard deviation (SD). f and g means non assigned family and genus.

### RNA:DNA ratios

The ratio between RNA and DNA derived relative proportions of certain microorganisms can be taken as a measure of their relative activity in the sample^31^. The RNA:DNA ratio at the phyla level is presented in Supplementary Table S3 for the more dominant phyla only since calculating ratios amongst the rare phyla may provide erroneous high ratios and division by zero. RNA:DNA ratios larger than one suggest a higher RNA deduced relative proportion than estimated from the DNA relative proportion. Overall, Cyanobacteria were the most active organisms (all RNA:DNA >1) followed by Gemmatimonadetes and the Deinococcus-Thermus phylum. Cyanobacterial RNA-deduced relative proportion was higher in all stations and seasons compared to the DNA-deduced relative proportion, while Gemmatimonadetes had a higher RNA-deduced relative proportion in autumn for all stations and in the Tidal station at all seasons. Deinococcus-Thermus activity was highest in the Tidal samples except for the autumn sample but instead was higher in the autumn Intermediate station. Some of the other phyla had a relative higher RNA relative proportion than DNA relative proportion in the summer Tidal station (Actinobacteria, Proteobacteria, and Planctomycetes) or in the autumn Dune station (Fusobacteria and Chlorobi). Finally, when comparing all RNA:DNA ratios, most phyla had their highest RNA:DNA ratio in autumn with the exception of Deinococcus-Thermus, Proteobacteria, Planctomycetes, and Bacteroidetes that had the highest relative activity in summer (Supplementary Table S3).

### Site- and season-specific cyanobacterial ecotypes

**Figure 4:**
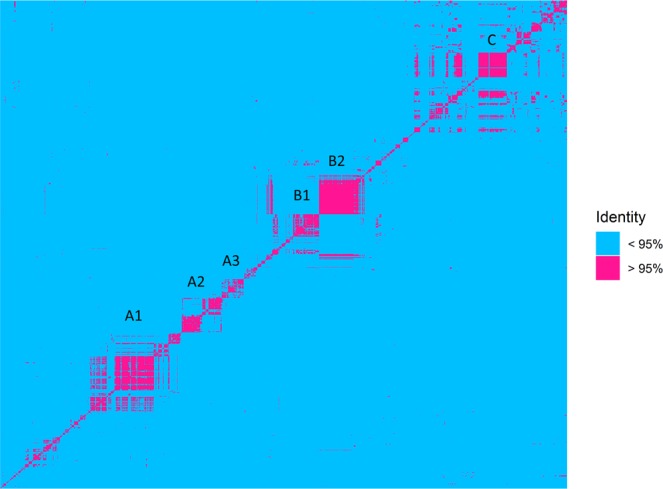
Heat map of the unique OTUs at 95% (or higher) identity (red) and less than 95% (blue). Clusters A1, A2, A3, B1, B2 and C were the retrieved OTUs for ecotypes analysis.

**Figure 5:**
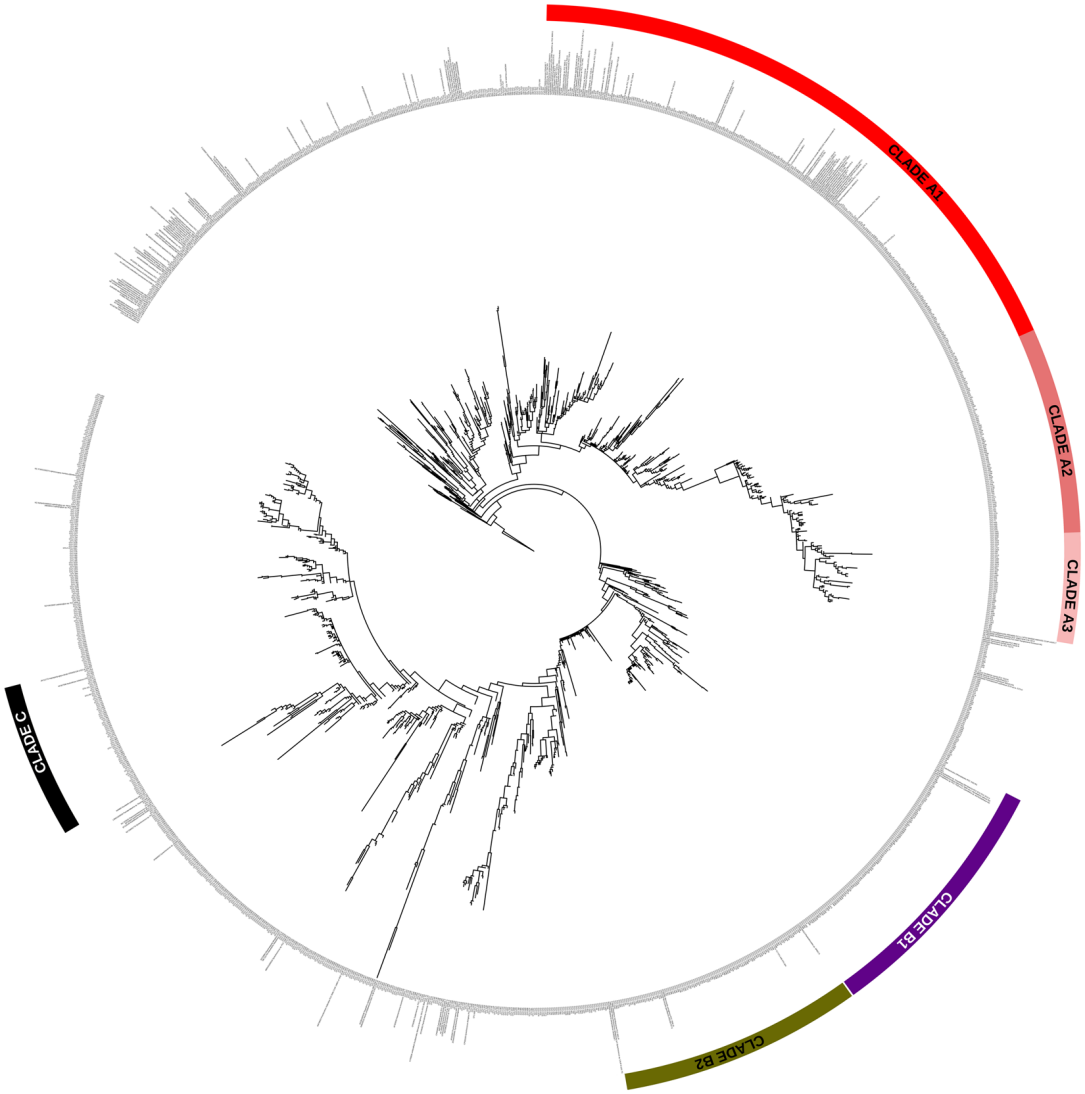
Maximum likelihood phylogenetic tree of the cyanobacterial ecotypes.

In order to identify cyanobacterial ecotypes that are specific for one station or one season the raw reads of the forward sequences annotated as belonging to the Cyanobacteria were further analysed. The resulting reads after high quality trimming were 275 nt long and spanned the V3 variable region. In total, 1.374 unique cyanobacterial OTUs were identified consisting of 142.944 reads (Supplementary Table S4). Of these, 667 OTUs (135.734 reads) clustered in 6 major clades that shared within each clade at least 95% sequence identity over the 275-base pair region (Figs 4 and 5). Each OTU within a clade was considered an ecotype of the same species. This identity threshold is well above the average identity of 86.6% for this region between all cyanobacterial OTUs found in this study and the 86% identity in this region calculated for known cyanobacterial species in the database. Taxonomic assignment of these ecotypes did not always provide the same best blast hit for each OTU and we therefore named the ecotypes after the most abundant identified genus in that clade. A total of 226 OTUs belonged to the A1 - *Nodularia* ecotype, 97 OTUs to A2 –*Kryptousia*, A3 and B3 were both assigned as *Halomicronema* sp. with 53 and 112 OTUs, respectively, 110 OTUs to B2 – *Coleofasciculus*, and 69 to C – *Crocosphaera* (Supplementary Table S4). The number of different *Nodularia* ecotypes was 25 to 15 times higher in spring than in summer and autumn respectively (Supplementary Table S5). The majority of these spring ecotypes, 89, was found only in spring. In contrast, the *Coleofasciculus* ecotype consisted of nearly 7 times more OTUs in autumn (96, including 33 unique ecotypes) compared to the summer (14) and none were found in spring (Supplementary Table S5). Ecotype A3 and B1 *Halomicronema* was also lowest in spring. The majority, 44, of *Crocosphaera* OTUs was present in autumn. In the B1 – *Halomicronema* ecotype only 5 out of 112 OTUs were unique for one season (summer) (Supplementary Table S6).

Spatial separation was found for 96 ecotypes of B1 – *Halomicronema* in the Intermediate station (Supplementary Table S7) of which only 5 were restrictedly found in this station (Supplementary Table S8). Of the B2 – *Coleofasciculus* ecotypes, the majority (90) were found in the Tidal station of which 21 were unique for this station (Supplementary Table S8). The other ecotypes were more evenly distributed over the sites. Of the A1 – *Nodularia* clade, 19 unique ecotypes were found that were confined to the Tidal station alone (Supplementary Table S8). Finally, 17 ecotypes belonging to A1-*Nodularia* and 21 belonging to B2 – *Coleofasciculus* were uniquely found in only one station and in only one season, Tidal spring and Tidal autumn respectively (Supplementary Table S9).

## Discussion

Spatiotemporal variations in relative proportion of active and resident members of coastal microbial mat communities was assessed through sequence analysis of the 16S rRNA molecule and its coding gene, respectively. Mats were sampled at three sites along a tidal gradient taking multiple samples at each site in order to account for local heterogeneity. This was repeated three times during a year to account for seasonality. Both spatial and seasonal aspects resulted in significant differences in microbial community composition and revealed insight in the seasonal ecological succession of different microorganisms in the three microbial mats (Fig. 2).

### Composition of the mat community along the tidal gradient

Salinity has been considered to be the most important determinant of community composition among microbial ecosystems worldwide^32^. The differences between the microbial mats along the tidal gradient correlated with the natural salinity gradient ranging from nearly freshwater close to the dunes to full seawater close to the low water mark^15^. Between those two extremes, the beach continuously experiences large fluctuations in salinity during the day and throughout the year as the result of irregular submersion, desiccation, and rainfall and resulting in the development of three major mat types: ‘Dune’, ‘Intermediate’, and ‘Tidal’^11,17,18^.

Multiple samples taken within one station, revealed the extant microheterogeneity within the mat communities (Fig. 2)^14^. Despite this microheterogeneity, cluster analysis of the samples revealed distinct communities per station that distinguished the microbial mats at the different sites along the tidal gradient (Fig. 2). The overall composition of the Dune mat was more similar to the Intermediate station than to the Tidal station, which emphasizes that the fully marine site is fundamentally different from the more terrestrial, low salinity sites.

Taxonomic annotation was based on the latest release of the Silva reference database, even when the proposed re-classifications still need a more solid basis. Silva database release 132 drastically changed since our previous work with database 111^14^. In the Proteobacteria, class Epsilonproteobacteria is now a phylum of their own and called Epsilonbacteraeota, while the class Betaproteobacteria is now seen as an order of Gammaproteobacteria (https://www.arb-silva.de/documentation/release-132/). Also, in the new release cyanobacterial genera have now received names, which makes it easier to compare them and refer to published work.

Cyanobacteria dominated both the DNA- and RNA-derived samples, which was expected, considering their central role as the colonizers of the beach and as the primary producers on which the microbial community relies (Fig. 3). None of the dominant genera of cyanobacteria appeared to be restricted to a specific station or season since they were present in all samples. A few (32) rare genera of cyanobacteria appeared to be absent at certain sites but they may have been missed due to their low relative proportion. Hence, neither season nor the tidal gradient appeared to be selective against any of the cyanobacteria.

Among the cyanobacterial ecotypes, 3 OTUs were restricted to the Dune station, 16 to the Intermediate station, and 41 to the Tidal station (Supplementary Table S8). The increasing number of unique cyanobacterial ecotypes from Dune to Tidal station emphasises the fundamental difference between the marine station and those higher up in the littoral. The marine station is not necessarily more diverse than the other mats (Table 1). Higher salinities (>70 g/kg = 2 times seawater) usually lowers microbial diversity^33,34^.

Comparison of RNA:DNA ratios are difficult due to amongst others differences in copy number of the 16S rRNA gene^35^, but ribosomal RNA can be used as a proxy for the activity of the corresponding microorganism^14^. RNA is associated with protein synthesis and therefore active organisms tend to have a higher RNA:DNA^36,37^. The RNA:DNA determined in this study show that seven of the fourteen abundant phyla have a higher activity in the Tidal station and five phyla were more active in the Dune station (Supplementary Table S3). Potentially, the tides result in a continuous influx and efflux of nutrients while the wave action disturbs the top sediment layer, which requires a more active, continuously growing population of especially Gemmatimonadetes and Deinococcus-Thermus. In the Dune station a dynamic interaction with the extant vegetation might require higher activity of amongst other cyanobacteria, Fusobacteria, and Chlorobi. Chloroflexi is the only phylum with a relatively higher active fraction in the intermediate station (Fig. 3). The intermediate station is less frequently affected by the tides, while vegetation is nearly absent leading to a potential stable community slowly developing community (Fig. 1).

Despite the different techniques used and the many years between sampling, the phyla composition of the microbial mats did not show major differences with our previous work^11^. The only disagreement with the present work was the very low relative proportion of Cyanobacteria and the absence of Betaproteobacteria in the Tidal station. The mats at this station might have developed further and became more similar to the mats higher in the littoral than was the case during the earlier studies.

### Seasonal community variations

Seasonal changes in microbial composition are indicative for the yearly development and destruction of the coastal microbial mats^7^. Seasonal succession in the resident and active coastal mat communities was followed by observing the decrease or increase of the number of taxonomic units (Supplementary Table S1). In addition, several taxa were more abundant in summer and formed summer climax populations. Overall, communities along the beach started with low diversity in spring and became more diverse during summer and autumn. This was most pronounced in the Dune station where the estimated richness increased during the year with 250 OTUs (448 genera) and the Shannon diversity index increased from 3.73 to 5.03. Richness also increased in the Intermediate and Tidal stations but diversity was highest in summer. Although the number of estimated (and observed) OTUs was lower in summer than in autumn, they were more evenly distributed over the different taxa in summer (Table 1). These results confirmed those of an earlier study of the same ecosystem^11,38^.

Genera like *Nodularia*, *Loktanella*, *Lyngbya*, and *Algoriphagus* that dominate in spring and decrease later in the year are likely initial colonizers of the beach and initiate mat formation. Some members of these genera are known to be potential phototrophic microorganisms that might have little nutritional requirements compared to chemoheterotrophs^39^. Photoautotrophs use inorganic carbon which is fixed at the expense of sunlight. Many cyanobacteria and anoxygenic Rhodobacterales are also capable of fixing atmospheric dinitrogen, giving them access to the second-most important element^40^.

The oxygenic photoautotrophic cyanobacteria initiate the formation of a coastal microbial mat by enriching the sediment with essential nutrients and provide structure and stability, which provides a habitat and niche for other functional groups of microorganisms^7^. Although the eukaryotic diatoms also play a role as primary producers and colonizers in microbial mats^41^, this study focuses on the bacterial component. Early colonizing Cyanobacteria include the diazotrophic *Nodularia* and *Lyngbya*, heterocystous and filamentous non-heterocystous, filamentous genera, respectively. *Nodularia* was especially abundant in spring^42^. This genus was less abundant during summer and autumn, when the mat had already been enriched with bound nitrogen and the mat developed extensive anoxic layers below the cyanobacterial mat. *Coleofasciculus*, a non-heterocystous cyanobacterium that is globally known as a microbial mat builder typically takes over as a dominant organism in these mature microbial mats and exude copious amounts of extracellular polymeric substances (EPS) that form the matrix in which the microorganisms are embedded^43^. EPS also increase the erosion threshold of the mat and provide a physically stable environment. Although *Coleofasciculus* seems to possess the genetic capacity for dinitrogen fixation, it is not certain if this organism actually does fix N_2_, but it would certainly require anoxic conditions for it^11^. The detection of the non-heterocystous diazotrophic cyanobacterium *Trichodesmium* as a colonizer in these coastal mats surprises and demands an explanation. *Trichodesmium* is known from the plankton in the (sub)tropical ocean and has never been reported from benthic environments such as microbial mats nor from temperate or cold regions. *Trichodesmium* may be morphologically and genetically confused with *Lyngbya*. We speculate that there may be a benthic form of *Trichodesmium* as is for instance the case with *Nodularia*, which also has a benthic (as in the mats studied here) and a planktonic ecotype (as in the Baltic Sea). Isolation of this supposed benthic ecotype of *Trichodesmium* will be necessary to prove this hypothesis.

Cyanobacterial ecotype distribution suggests a high diversification in spring with 205 different *Nodularia* ecotypes (Supplementary Table S5). The mature summer mats are characterized by the multi-layer organization and the physicochemical micro-gradients. This creates a plethora of habitats and niches for various ecotypes of successor Cyanobacteria such as *Halomicronema* in summer and *Coleofasciculus* in autumn.

Several members of Rhodobacterales, notably *Loktanella*, are abundant in developing mats while in low numbers in mature mats (supplementary Table S1). Rhodobacterales are metabolically versatile bacteria capable of aerobic and anaerobic respiration, anaerobic fermentation, sulphur oxidation^44,45^, autotrophic carbon fixation, nitrogen fixation, and hydrogen production^40^, which gives them a central role in the microbial mat ecosystem. Members of this family dominate surface microbial communities in the English Channel during spring, which was attributed to their preference for low nutrient concentrations^46^. The precise role of *Loktanella* in the mats remains to be determined. *Loktanella* belongs to the *Roseobacter* clade that is known to be equipped with a plethora of different metabolic capabilities^47^. Potential sulphur oxidation by members of the Rhodobacterales would require the production of sulphide by sulphate reducers^48^, notably Desulfobulbaceae as found in this study. Although the relative proportion of these sulphate reducing bacteria in the microbial mats is low (<0.5%) their activity may nevertheless be sufficient to provide sulphide and establish anoxia in the lower parts of the mat. Successors of the colonizing sulphate reducers are Desulfobacteraceae, which increased from 0.4% in spring to 0.6% in autumn (supplementary Table S1).

Early colonizing Bacteroidetes of the genera *Algoriphagus*, *Bizionia*, and *Lewinella*, are aerobic heterotrophs that may degrade high molecular polymeric substances such as alginates^49–51^. *Loktanella*, *Algoriphagus*, and *Bizionia* are known from Antarctic microbial mats^44^. The colonizing species in the Dutch coastal mats may therefore also be cold-adapted. Similarly, the cyanobacterium *Nodularia* may have originated from a cold-adapted branch within the Nostocales^52^.

In addition to the cyanobacterial successors that dominate the summer and autumn communities, certain heterotrophic bacteria out-compete the colonizing microorganisms. These heterotrophic successors may grow faster at the summer temperatures and benefit from the excess of nutrients. Moreover, more micro-habitats may have been formed due to the deposition of EPS and the lamination of the microbial mat with physicochemical gradients of amongst others light, oxygen, pH, and sulphide^53^. These micro-habitats provide niches for specialist microorganisms for example for the degradation of polymers and the utilization of fermentation products^54^.

In autumn, the Dune station is characterized by a higher contribution of Proteobacteria, Epsilonproteobacteria, Bacteroidetes, Chloroflexi, Actinobacteria, and Firmicutes when compared to the Tidal mat (Fig. 2). This difference may have been caused by the presence of plant-derived organic matter. Some of these Proteobacteria may have specialized in the aerobic and anaerobic decomposition of this plant-derived material^33^.

The higher relative proportion of Chloroflexi, Actinobacteria, Firmicutes, and Deinococcus-Thermus and the lower relative proportion of Cyanobacteria in the Dune station in autumn may be caused by the salt-marsh vegetation. Chloroflexi were dominated by a novel genus of the family of Anaerolineaceae and represented the third most abundant genus in these mats (Fig. 2). The family of Anaerolineaceae is known for syntrophic relationships with methanogenic Archaea^55^, which are known to be present in the Dune mats^11^. These methanogens may benefit from decaying plant material through a syntrophic relationship with Anaerolineaceae^56–58^. The ecological role of the common soil bacteria Gemmatimonadetes is not precisely known, but our studies showed that they become more active in autumn. Gemmatimonadetes prefer dry over wet soils^59^ and have an highest relative proportion in the late summer^60^. In our study, Gemmatimonadetes activity was most pronounced in the tidal station. Hence, their role in the coastal microbial mats remains unexplained. None of the identified phyla revealed higher activity in spring.

## Conclusions

Coastal microbial mats are unique ecosystems with a dynamic spatial temporal composition revealing distinct seasonality, although the resident fraction may be perennial. The observed seasonality resembles the succession of different vegetation types after colonizing the beach sand.

This study shows seasonality in the studied coastal microbial mats, especially for the active mat microorganisms. At any time of the year, any of the three stations host different communities. However, independent of the station or the season, three phyla dominate the mats: Cyanobacteria, Proteobacteria, and Bacteroidetes. Potentially cold-adapted members of the cyanobacteria and *Loktanella* sp. colonize the beach sediment, and are subsequently followed by more competitive, opportunistic heterotrophic microorganisms. The climax stage of the intermediate coastal mat community is characterized by the well-studied high-diversity, layered micro-scale ecosystem. Although the physicochemical characteristics of the mats along the tidal gradient are obviously different, the mats are nevertheless composed of the same main groups of microorganisms that may only differ by their ecotypes to optimally fulfil their functions in the different mat types.

## Supplementary information

Dataset 1
