# Supplementary material for: Seasonal development of a coastal microbial mat
Source: Sci Rep. 2019 Jun 21;9:9035. doi: 10.1038/s41598-019-45490-8 (PMC6588573; doi:10.1038/s41598-019-45490-8)
Supplement: Supplementary file 1 — Dataset 1 [file 41598_2019_45490_MOESM1_ESM.pdf]

Seasonal development of a coastal microbial mat

Daniela Clara Cardoso[1], Mariana Silvia Cretoiu[1,3], Lucas J. Stal[1,2], Henk Bolhuis[1*]

1) Department of Marine Microbiology and Biogeochemistry, Royal Netherlands Institute for Sea Research, and Utrecht University, Den Hoorn, the Netherlands.
2) Freshwater and Marine Ecology (IBED-FAME), University of Amsterdam, Amsterdam, The Netherlands.
3) Current address: Bigelow Laboratory for Ocean Sciences, East Boothbay, ME 04544, USA

*) Correspondence should be addressed to henk.bolhuis@nioz.nl

Supplementary Information

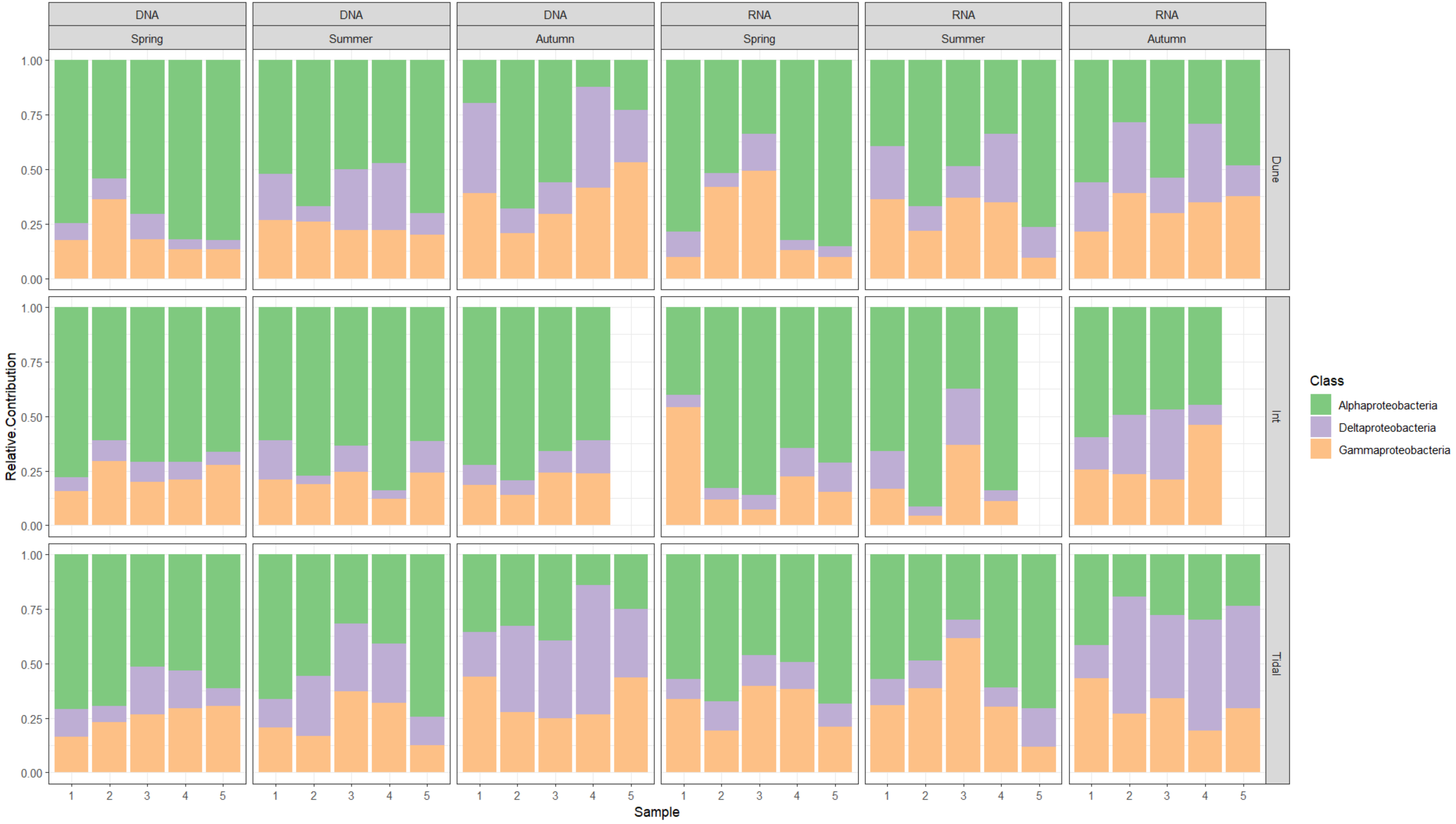

Figure S1. Relative contribution of the proteobacterial orders. Sampling type (RNA, DNA) and sampling season are indicated on top. The sampling station is indicated in the right-side bar. Samples taken from the same station at the same time are indicated in the horizontal axis.

Supplementary Table S1: Seasonal increase, decrease and summer peak of the most abundant genera for the resident fraction (DNA).

**Seasonal increase**	**% Spring**	**SD**	**% Summer**	**SD2**	**% Autumn**	**SD3**
Coleofasciculus	0.12	0.11	2.14	2.57	14.03	25.08
SBR1031_f_*g*	0.07	0.06	0.21	0.15	1.65	4.60
Sulfurimonas	0.39	0.45	0.13	0.18	8.06	12.05
A4b_*g*	0.07	0.05	0.66	0.49	1.16	1.99
Truepera	0.19	0.25	1.06	1.50	2.04	5.57
Balneolaceae_g	0.10	0.15	0.73	0.79	0.88	2.30
Desulfobacteria_*g*	0.14	0.10	0.69	0.59	1.09	1.67
Anaerolineaceae_*g*	0.29	0.31	0.59	0.71	1.09	2.17
Phormidesmis	0.47	0.43	1.05	1.28	1.59	1.82
Rivularia	0.84	1.00	0.54	0.61	1.84	2.78

**Seasonal decrease**	**% Spring**	**SD**	**% Summer**	**SD2**	**Autumn**	**SD3**
Nodularia	16.36	8.39	0.72	1.05	0.94	1.62
Algoriphagus	2.39	1.11	0.35	0.45	0.27	0.32
Nostocaceae_*g*	1.04	0.82	0.27	0.50	0.18	0.28
Lewinella	1.39	0.64	0.64	0.42	0.31	0.25
Bizionia	2.30	1.67	0.68	0.80	0.67	0.82
Loktanella	12.00	6.19	7.75	6.50	3.69	3.27
Lyngbya	2.89	3.85	1.04	1.20	0.93	1.09
Congregibacter	2.14	1.45	0.96	0.76	0.85	1.10
Flavobacteriaceae_*g*	1.81	1.28	1.20	1.47	0.89	1.43
Rhodobacteraceae_*g*	1.16	1.06	0.78	0.60	0.65	0.61

**Summer peak**	**% Spring**	**SD**	**% Summer**	**SD2**	**% Autumn**	**SD3**
Leptolyngbya	0.08	0.11	6.13	9.47	0.59	0.97
Psychroflexus	0.14	0.14	2.90	2.50	0.07	0.09
Nodosilineaceae_*g*	0.26	0.19	4.29	5.82	2.48	4.71
Halomicronema	0.35	0.21	1.93	2.01	0.54	0.75
Phormidium	0.28	0.25	1.35	1.32	0.91	1.30
Trichodesmium IMS101	0.84	1.26	2.64	3.32	0.13	0.28
Trichodesmium IMS101_*g*	0.41	0.26	0.96	0.72	0.42	0.28
Halochromatium	0.80	0.97	1.50	1.32	0.32	0.36
Muriicola	0.57	0.72	0.91	0.96	0.22	0.34

Supplementary Table S2: Seasonal increase, decrease and summer peak of the most abundant genera for the resident fraction (RNA).

SEASONAL INCREASE	%SPRING	SD	% SUMMER	SD	% AUTUMN	SD
Sulfurimonas	0.03	0.04	0.02	0.05	1.23	2.97
Coleofasciculus	2.33	3.85	15.95	16.61	32.80	34.09
Bacteroidetes vadinha17_*g*	0.03	0.03	0.01	0.02	0.34	0.59
Pauc26f	0.02	0.02	0.08	0.13	0.27	0.36
Bryobacter	0.03	0.04	0.20	0.54	0.29	0.46
Desulfobacteraceae_*g*	0.06	0.06	0.36	0.49	0.60	0.59
Rd017_*f*_*g*	0.10	0.12	0.44	1.05	0.75	1.67
Candidatus competibacter	0.04	0.03	0.07	0.08	0.23	0.42
Spirulina p7	0.07	0.13	0.06	0.17	0.42	0.60
Phormidesmis	0.31	0.30	0.45	0.66	1.81	1.77

SEASONAL DECREASE	% SPRING	SD	% SUMMER	SD	AUTUMN	SD
Limnothrix	0.49	0.83	0.01	0.02	0.03	0.04
Symphothece	0.44	0.42	0.10	0.06	0.03	0.04
Nodularia	36.70	19.88	6.64	15.06	2.77	3.51
Nostocaceae_*g*	4.01	2.89	0.89	1.32	0.69	0.65
Xuhuaishuia	0.23	0.08	0.19	0.21	0.05	0.04
Candidatus electrothrix	0.38	0.41	0.22	0.48	0.10	0.18
Calothrix	0.25	0.37	0.05	0.16	0.09	0.16
Loktanella	1.63	0.82	1.52	1.41	0.58	0.57
Aphanizomenon	0.29	0.38	0.07	0.18	0.10	0.17
Chromatiaceae_*g*	0.19	0.28	0.10	0.18	0.07	0.15

SUMMER PEAK	% SPRING	SD	% SUMMER	SD	% AUTUMN	SD
Xanthomonadaceae_*g*	0.001	0.002	0.723	2.458	0.017	0.031
Rapidithrix	0.003	0.007	0.306	0.748	0.003	0.006
Leptolyngbya	0.092	0.069	3.606	6.022	1.374	1.433
Arthrospira	0.009	0.010	0.175	0.424	0.128	0.134
Phormidesmiaceae_*g*	0.024	0.039	0.442	0.991	0.108	0.136
Sandaracinaceae_*g*	0.017	0.015	0.241	0.177	0.142	0.118
Aquiflexum	0.021	0.031	0.271	0.888	0.063	0.077
Woeseia	0.037	0.046	0.454	0.770	0.108	0.177
Unassigned	0.062	0.066	0.704	1.484	0.132	0.075
Wilmottia	*0.064*	*0.068*	*0.572*	*0.885*	*0.272*	*0.509*

Supplementary table S3: RNA:DNA ratio at the phylum level

Phyla	Spring			Summer			Autumn		
	Dune	Intermediate	Tidal	Dune	Intermediate	Tidal	Dune	Intermediate	Tidal
Cyanobacteria	2,883	2,734	2,192	2,425	2,881	1,459	4,798	1,799	1,824
Gemmatimonadetes	0,642	0,515	1,032	0,755	0,297	3,716	2,398	2,194	2,902
Deinococcus-Thermus	0,288	0,647	3,137	0,371	0,162	7,436	0,260	1,142	0,592
Actinobacteria	0,123	0,227	0,766	0,648	0,135	1,028	0,817	0,608	0,463
Proteobacteria	0,303	0,282	0,554	0,552	0,320	1,157	0,707	0,382	0,382
Fusobacteria	0,048	0,000	0,000	0,008	0,117	0,197	3,532	0,000	0,044
Planctomycetes	0,096	0,215	0,147	0,181	0,058	1,883	0,122	0,793	0,298
Acidobacteria	0,175	0,149	0,234	0,220	0,190	0,946	0,389	0,627	0,697
Chlorobi	0,268	0,106	0,149	0,131	0,121	0,089	1,731	0,234	0,127
Spirochaetes	0,236	0,126	0,164	0,060	0,104	0,325	0,628	0,535	0,181
Bacteroidetes	0,077	0,083	0,114	0,798	0,080	0,225	0,240	0,180	0,225
Chloroflexi	0,087	0,095	0,105	0,205	0,207	0,141	0,123	0,575	0,088
Verrucomicrobia	0,018	0,012	0,029	0,062	0,018	0,097	0,067	0,086	0,034
Firmicutes	0,020	0,046	0,075	0,032	0,013	0,036	0,023	0,111	0,038

Red box indicates phyla that are more abundant in the RNA fraction than in the DNA fraction

Only abundant phyla were included

Supplementary table S4: Unique ecotypes and correspondent number of reads

Species	# ecotype	# reads	% ID[1]
A1 - *Nodularia sp.*	226	45146	96.7
A2 - *Kryptousia sp.*	97	18833	92.1
A3 - *Halomicronema sp.*	53	3163	94.0
B1 - *Halomicronema sp.*	112	27386	94.8
B2 - *Coleofasciculus sp.*	110	8558	98.8
C - *Crocosphaera sp.*	69	32648	92.9
Total	667	135734	

[1] average % identity with known species

Supplementary table S5: Number of ecotypes per season

species\season	spring	summer	autumn	Total
A1 - *Nodularia* sp.	205	8	13	226
A2 - *Kryptousia* sp.	56	27	14	97
A3 - *Halomicronema* sp.	6	24	23	53
B1 - *Halomicronema* sp.	3	67	42	112
B2 - *Coleofasciculus* sp.	0	14	96	110
C - *Crocosphaera* sp.	17	8	44	69

Supplementary table S6: Number of ecotypes unique for only one season

species\season	spring	summer	autumn	Total
A1 - *Nodularia* sp.	89	3	1	93
A2 - *Kryptousia* sp.	22	2	0	24
A3 - *Halomicronema* sp.	0	24	23	47
B1 - *Halomicronema* sp.	0	5	0	5
B2 - *Coleofasciculus* sp.	0	0	33	33
C - *Crocosphaera* sp.	0	0	1	1

Supplementary table S7: Number of ecotypes per station

Species/station	Dune	Intermediate	Tidal	Total
A1 - *Nodularia sp.*	38	97	91	226
A2 - *Kryptousia sp.*	56	27	14	97
A3 - *Halomicronema sp.*	18	24	11	53
B1 - *Halomicronema sp.*	12	96	4	112
B2 - *Coleofasciculus sp.*	6	14	90	110
C - *Crocosphaera sp.*	8	18	43	69
				667

Supplementary table S8 Number of ecotypes unique for one station

species\station	Dune	Intermediate	Tidal	Total
A1 - *Nodularia* sp.	0	5	19	24
A2 - *Kryptousia* sp.	3	6	0	9
A3 - *Halomicronema* sp.	0	0	1	1
B1 - *Halomicronema* sp.	0	5	0	5
B2 - *Coleofasciculus* sp.	0	0	21	21
C - *Crocosphaera* sp.	0	0	0	0
	3	16	41	

Supplementary table S9: Number of ecotypes found in only one station and one season

Species	Unique ecotypes	
A1 - *Nodularia sp.*	17	Tidal spring
A2 - *Kryptousia sp.*	0	
A3 - *Halomicronema sp.*	1	
B1 - *Halomicronema sp.*	2	
B2 - *Coleofasciculus sp.*	21	Tidal autumn
C - *Crocosphaera sp.*	0	
